# Development of a biological at-risk volume using apparent diffusion coefficient for parotid-sparing radiation therapy planning

**DOI:** 10.1093/bjro/tzaf020

**Published:** 2025-08-13

**Authors:** Katelyn Cahill, Catriona Hargrave, Patrick O’Connor, Mark Denham, Nathan Hearn, Dinesh Vignarajah, Zack Y Shan, Myo Min

**Affiliations:** Adem Crosby Centre—Radiation Oncology, Sunshine Coast University Hospital, Birtinya, QLD 4575, Australia; Thompson Institute, University of the Sunshine Coast, Birtinya, QLD 4575, Australia; Radiation Oncology Princess, Alexandra Hospital Raymond Terrace, Brisbane, QLD 4101, Australia; Faculty of Health, School of Clinical Sciences, Queensland University of Technology, Brisbane, QLD 4000, Australia; Adem Crosby Centre—Radiation Oncology, Sunshine Coast University Hospital, Birtinya, QLD 4575, Australia; School of Information Technology and Electrical Engineering, University of Queensland, St Lucia, QLD 4072, Australia; Department of Medical Imaging, Sunshine Coast University Hospital, Birtinya, QLD 4575, Australia; Thompson Institute, University of the Sunshine Coast, Birtinya, QLD 4575, Australia; Department of Medical Imaging, Sunshine Coast University Hospital, Birtinya, QLD 4575, Australia; Adem Crosby Centre—Radiation Oncology, Sunshine Coast University Hospital, Birtinya, QLD 4575, Australia; School of Medicine and Dentistry, Griffith University, Sunshine Coast Health Institute, Birtinya, QLD 4575, Australia; Thompson Institute, University of the Sunshine Coast, Birtinya, QLD 4575, Australia; Adem Crosby Centre—Radiation Oncology, Sunshine Coast University Hospital, Birtinya, QLD 4575, Australia; Thompson Institute, University of the Sunshine Coast, Birtinya, QLD 4575, Australia; School of Medicine and Dentistry, Griffith University, Sunshine Coast Health Institute, Birtinya, QLD 4575, Australia

**Keywords:** apparent diffusion coefficient, diffusion-weighted MRI, biological at-risk volume, head and neck cancer, parotid gland, radiotherapy

## Abstract

**Objectives:**

Xerostomia toxicity continues to contribute towards a decrease in quality of life in head and neck cancer patients. Diffusion weighted MRI and the associated apparent diffusion coefficient (ADC) may identify the radiosensitive region within the parotid gland (PG). This study retrospectively assesses the feasibility of using percentile threshold values from the ADC map to generate a biological at-risk volume (BRV). The location and distribution of BRV are evaluated across the PG.

**Methods:**

Image registration between the planning CT and MRI-simulation images was performed and reviewed to ensure accurate translation of ADC data when contouring the PG. Histogram analysis was undertaken using the 20th, 30th, and 50th percentile ADC values of each contoured PG to form the BRV. The whole PG was split into 8 anatomical sectors at a common intersection point to evaluate the distribution of BRV throughout.

**Results:**

The BRV distribution for each percentile was mapped across the whole contoured PG and each anatomical sector contour. The largest distribution was predominantly found in the superolateral sectors.

**Conclusions:**

The 20th and 30th percentile ADC values can be used to form a BRV of the PG. The location of the BRV distribution indicates a potential relationship between ADC thresholds and the functional region of the PG.

**Advances in knowledge:**

The BRV is located in a favourable position within the PG and could be used to further spare this salivary gland during dose optimization. The feasibility of this approach will be explored in a future retrospective dosimetry study.

## Introduction

The complexity of radiation therapy (RT) treatment optimization and delivery for head and neck cancer (HNC) is compounded by the proximity of adjacent organs at-risk (OAR) to the target volume. Advanced techniques such as intensity modulated radiotherapy (IMRT) and volumetric modulated arc radiotherapy (VMAT) have enhanced the optimization process, producing highly conformal dose to the target volume, in turn, reducing dose to neighbouring sensitive regions.^1^ This has allowed the development of parotid-sparing techniques which have seen a reduction in radiation induced complications.^2^ Despite such advancements, approximately 40% of HNC patients are still experiencing acute toxicities such as xerostomia.^3^^,^^4^ Subconscious actions such as swallowing, taste, and speech are impacted by xerostomia, this leads to a decrease in quality of life (QoL) in a group where incidence and survivorship is increasing.^5–7^ This indicates that further research is required to explore novel techniques that have the potential to be more effective in reducing xerostomia toxicities.

Preclinical investigations into the dose-volume relationship and dose distributions of the parotid gland in mice have shown that more effective parotid-sparing strategies may be possible by targeting region specific radiosensitivity within the salivary gland.^8^^,^^9^ Correlations have been made between the location of the excretory ducts and sensitivity of the parotid gland, indicating that the stem cells reside within the cranial region of the parotid gland.^10^ This is in accordance with van Luijk et al^11^ and more recently, van Rijn-Dekker et al^12^ who have shown that dose to the region of the parotid gland that contains the major ducts has been able to predict parotid gland dysfunction. With this knowledge, split organ delineation and regional parotid-sparing techniques of the superficial lobe have been trialled. Results have shown further reduction in dose and xerostomia severity which supports the stem cell location.^13–17^ These studies report on various methods for manual anatomical delineation to create a subregion to spare within the parotid gland. To our knowledge, delineation of a subvolume using an automatic approach, combined with functional MRI of the parotid gland has not yet been investigated.

Diffusion-weighted MRI (DW-MRI) has been used to evaluate salivary gland function, including predicting the severity of xerostomia during RT of the head and neck.^18^ This functional MRI sequence demonstrates the ability of water molecules to move freely within tissue through Brownian motion and is indicative of the cellularity of an area of interest.^19^ The rate of diffusion can then be quantified using the apparent diffusion coefficient (ADC) map using the signal change observed in the DW-MRI over 2 or more diffusion *b* values. The increased signal is represented by a low ADC value (mm^2^/s), indicating the inability of free motion of the water molecules.^20^ DW-MRI and the associated ADC map provides valuable insight into the microstructure of cells whereby restricted diffusion could indicate a hypercellular or functionally critical region.^21^ Historically, parotid gland radiosensitivity has been modelled on the mean dose delivered to the whole gland. However, this approach may overlook functionally heterogeneous subregions within the gland that are more susceptible to radiation induced damage. To address this limitation, we introduce the novel concept of a biological at-risk volume (BRV), a subvolume within the parotid gland identified through histogram analysis of voxel-based ADC values. This study aims to assess the feasibility of deriving BRVs from ADC percentile thresholds and to evaluate its spatial distribution across the entire parotid gland.

## Methods

### Patient selection

Ethics approval for this retrospective study was granted from the local health services human research ethics committee (HREC/2019/QPCH/53545). Eligibility criteria included patients who had undergone MRI-Simulation (MRI-Sim) with RT specific protocols and completed a radical course of RT for primary and/or post-operative bi-lateral HNC (C00.0—C14.8) between January 2021 and July 2022. Patients with post-operative parotid tumours were excluded.

### Image acquisition for planning

CT scans were acquired for treatment planning with 120 kV and 2.0 mm slice thickness on a SOMATOM CT scanner (Siemens Healthcare, Erlangen, Germany). MRI-Sim images were acquired on a 1.5 T MAGNETOM Aera (Siemens Healthcare, Erlangen, Germany) with an INSIGHT MRI Overlay (Qfix, Avondale PA, United States). INSIGHT MRI Coil Holders were used to position 2 large Flex 4 coils and a Body 18 long flex coil enabling replication of the treatment position with thermoplastic mask and neck and shoulder immobilization equipment from the planning CT scan. A 32-channel spine coil was used under the MRI Overlay. Acquired sequences include a turbo spin-echo T2-weighted, gradient-echo T1-weighted, and multi-shot echo-planar DW-MRI, where *b* values of 50, 400, and 800 (mm^2^/s) were used with a 4 mm slice thickness. ADC maps are generated automatically as part of standard DW-MRI acquisition. Imaging parameters have been established for routine MRI-Sim use, standard departmental quality assurance procedures were followed.^22^

### Image registration assessment

Automated rigid image registration was performed between the planning CT and MRI-Sim images using MIM Maestro (MIM Software Inc, Cleveland, OH, United States). In addition to this, the registration between the T2-weighted and DW-MRI sequences was also performed to ensure no gross motion occurred between acquisitions. These fusions were reviewed by two radiation therapists independently to ensure registration accuracy. For the purposes of this study, a retrospective quantitative assessment was also performed to evaluate the reproducibility of the parotid location between imaging modalities. Ipsilateral and contralateral parotid glands were manually contoured based off the planning CT (Parotid_pCT) and the T2-weighted MRI (Parotid_T2). The image registrations confirmed exact Digital Imaging and Communications in Medicine (DICOM) co-ordinates between MRI sequences. This provided confidence in using the Parotid_T2 contours to confirm the positioning accuracy of the ADC data, relative to the Parotid_pCT contour. These regions of interest (ROI) were quantitatively evaluated using dice similarity coefficient (DSC), mean distance to agreement (MDA), and Hausdorff distance (HD maximum and 95th percentile). These descriptive metrics were generated using MIM Maestro and 3D Slicer (http://www.slicer.org).^23^ Based on the tolerances recommended in Task Group 132,^24^ regions with a DSC >0.8 or an MDA and 95% HD <3 mm were considered appropriate for clinical use. This verification was deemed essential to be able to confirm the accurate transfer of ADC information from the ADC map to the planning CT.

### Delineation of apparent diffusion coefficient and BRV contours

**Figure 1.**
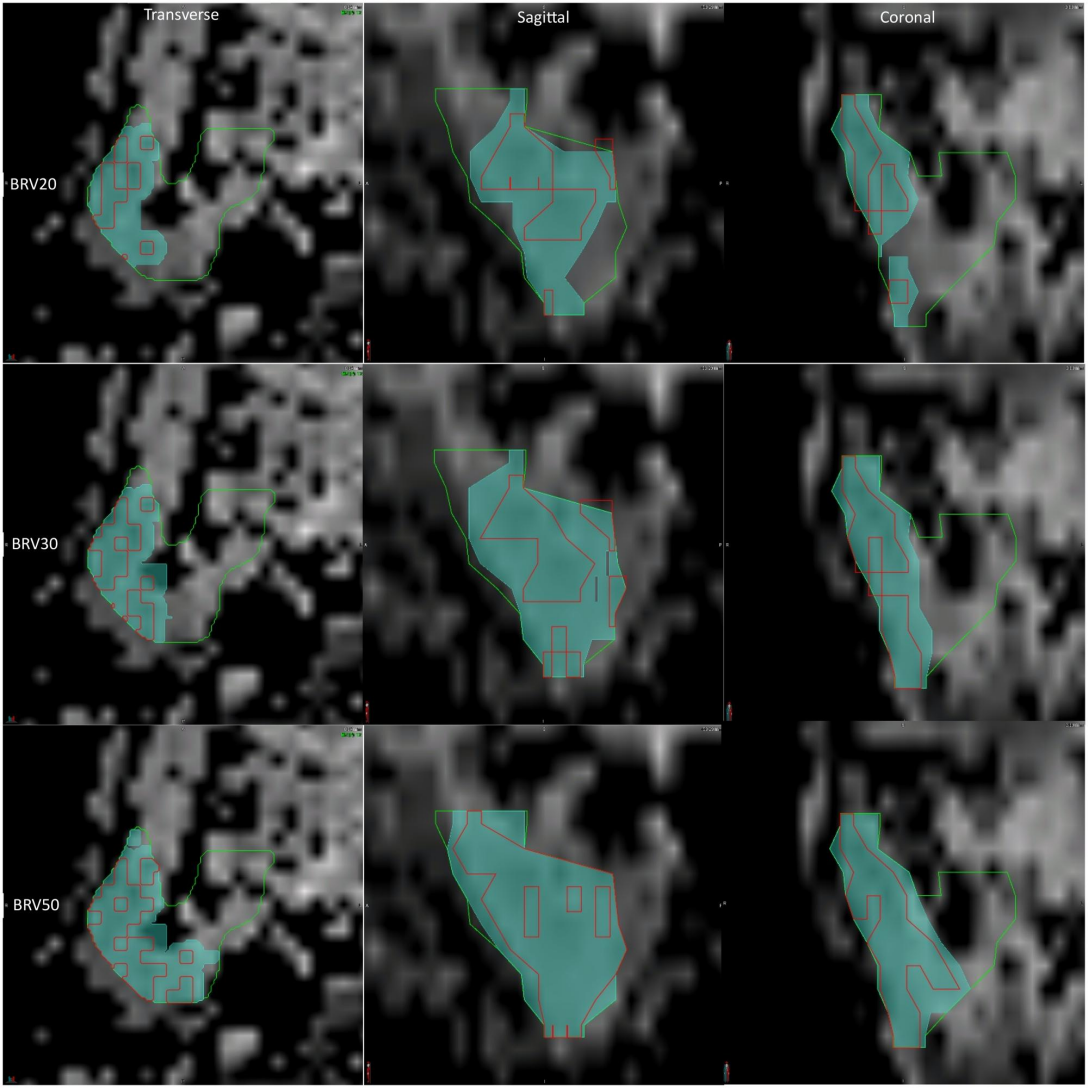
Biological at-risk volume (BRV) examples in the ipsilateral parotid gland. Twentieth percentile BRV (BRV20), 30th percentile BRV (BRV30), and 50th percentile BRV (BRV50) contours demonstrating the automated percentile apparent diffusion coefficient contour in red and the expanded BRV contour in cyan.

In contrast to previous studies that used manual anatomical subregion delineation, this study employed an automated ADC based process. The ADC derived BRV contours were generated in MIM Maestro (MIM Software Inc, Cleveland, OH, United States) using a department specific automated workflow. For each patient, histogram analysis was performed on the ipsilateral and contralateral Parotid_T2 ROI with the external carotid artery and retromandibular vein removed.^25^^,^^26^ The 20th, 30th, and 50th percentile ADC values were extracted from these cumulative histograms. For each selected percentile, the corresponding ADC value was defined on the ADC map and used to automatically generate a voxel-based contour. To minimize the inclusion of any ADC voxels arising from contouring variations, edge effects or adjacent non-parotid tissue, the lower ADC range was set to 5. This initial voxel-based contour was volumetrically cleaned to remove outlier voxels smaller than 0.5 cc. Subsequently, the resulting contour was uniformly expanded by 2 mm and clipped to the anatomical boundary of the Parotid_T2 ROI. The final delineated volumes, BRV20, BRV30, and BRV50 represent subvolumes within the ipsilateral and contralateral parotid glands corresponding to the 20th, 30th, and 50th percentile ADC thresholds, respectively (see Figure 1). The final BRVs were independently reviewed by a senior radiation therapist and senior radiation oncologist. This automated process improved overall BRV reproducibility and reduced any potential inter-observer variability.

### BRV distribution across anatomical parotid gland sectors

**Figure 2.**
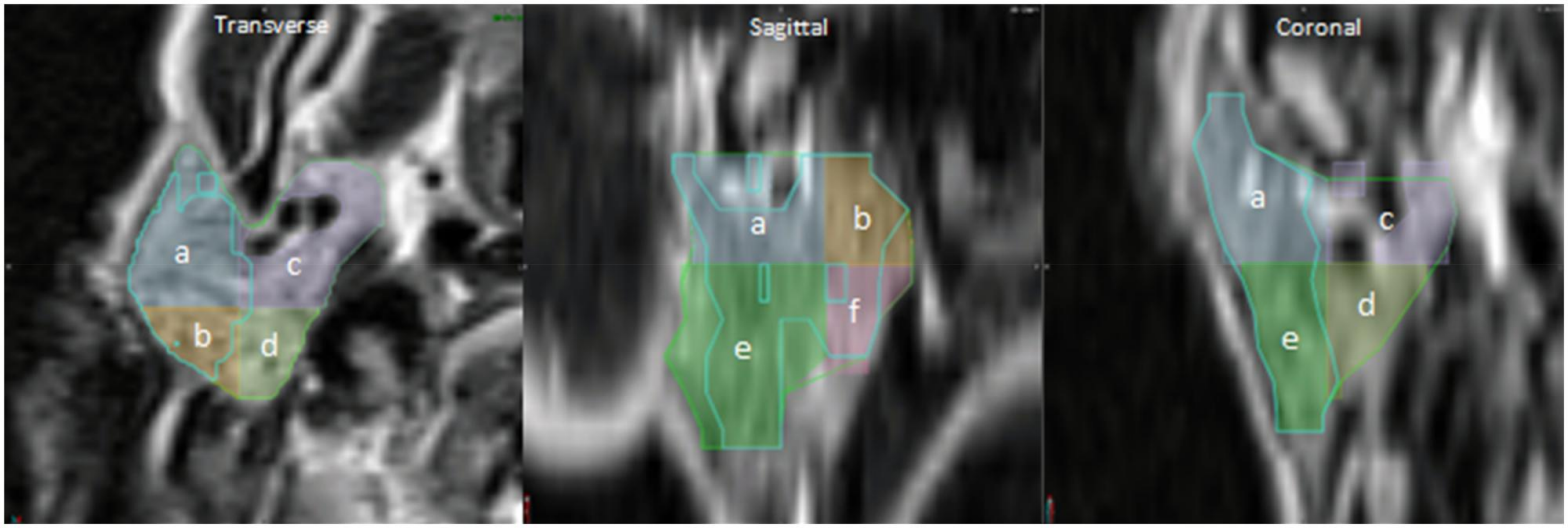
Example of anatomical sectors demonstrating the occupation of BRV30 (cyan contour) throughout the ipsilateral parotid gland. (a—SupAnteroLat; b—SupPosteroLat; c—SupAnteroMed; d—SupPosteroMed; e—InfAnteroLat; f—InfPosteroLat).

Given the emerging evidence of regional radiosensitivity within the parotid gland, each parotid gland was split into eight anatomical sectors to evaluate the spatial distribution of BRVs. Each sector was defined by anatomical orientation and labelled SupAnteroLateral, SupPosteroLateral, SupAnteroMedial, SupPosteroMedial, InfAnteroLateral, InfPosteroLateral, InfAnteroMedial, and InfPosteroMedial (Figure 2). The sector boundaries were formed at a common intersection point, based on anatomical landmarks. Cranial/caudal division occurred at the inferior level of the mastoid process, superficial/deep division was lateral to the retromandibular vein^13^ and anterior/posterior division was at mid-separation of the parotid gland in the transverse plane. Each BRV contour was then split into 8 sectors in line with the anatomical descriptions. This allowed for a descriptive analysis to determine where the largest BRV distribution was located per parotid sector. Statistical analysis was performed using Pearson correlation to determine if there is a spatial association between the BRV location and the corresponding ADC percentile thresholds.

## Results

### Quantitative analysis

#### Quantitative assessment of parotid gland reproducibility

Eleven consecutive patients were eligible and included for ADC analysis of the ipsilateral and contralateral parotid glands. Overall, there was minimal variation between parotid position in the planning CT and the T2-weighted MRI-Sim images (see Table 1). Although the mean HD max for the ipsilateral parotid is above 3 mm, the mean 95th percentile HD is a better representation of the similarity of the volumes which is shown to be within the guidelines.

**Table 1.** Summary of descriptive metrics for similarity of parotid gland contours between planning CT and T2-weighted MRI.

	Average descriptive metrics			
Parotid side	DSC	MDA (mm)	HD max (mm)	95% HD (mm)
Ipsilateral parotid	0.986	0.095	3.398	0.824
Contralateral parotid	0.985	0.091	3.540	0.969

#### Analysis of apparent diffusion coefficient and parotid gland BRV

The average of the median ADC values of the whole ipsilateral and contralateral parotid gland for all 11 patients was 1016 and 1004 mm^2^/s, respectively. As expected, the ADC value increased as the percentile threshold increased (Table 2). The median volume for the whole ipsilateral parotid was 24.37 cc and the whole contralateral parotid was 25.17 cc. As summarized in Table 3, the BRV size for each percentile threshold was comparable, with the contralateral parotid being on average 1.4 cc larger. The BRV20 occupied approximately 37% of the combined ipsilateral and contralateral parotid volumes, whereas the BRV30 occupied 54% and the BRV50 75%.

**Table 2.** Mean and range of the 20th, 30th, and 50th apparent diffusion coefficient values for whole, ipsilateral and contralateral parotid glands.

Parotid side		Mean ADC value (range) (mm^2^/s)
Ipsilateral parotid	Whole parotid	1016 (835-1160)
	20th percentile	853 (694-966)
	30th percentile	925 (751-1039)
	50th percentile	1060 (855-1201)
Contralateral parotid	Whole parotid	1004 (843-1161)
	20th percentile	836 (702-1004)
	30th percentile	909 (774-1075)
	50th percentile	1042 (882-1227)

**Table 3.** Median volume and range of whole parotid gland and biological at-risk volume of ipsilateral and contralateral parotids.

Parotid gland side		Median volume (range) (cc)
Ipsilateral parotid	Whole parotid	23.37 (7.52-41.85)
	BRV20	9.34 (2.21-12.99)
	BRV30	14.16 (2.66-5.41)
	BRV50	19.15 (5.41-24.98)
Contralateral parotid	Whole parotid	25.17 (11.35-41.92)
	BRV20	10.08 (3.91-16.72)
	BRV30	14.50 (5.8-23.71)
	BRV50	19.09 (8.28-32.90)

#### Distribution of BRV across parotid gland

**Figure 3.**
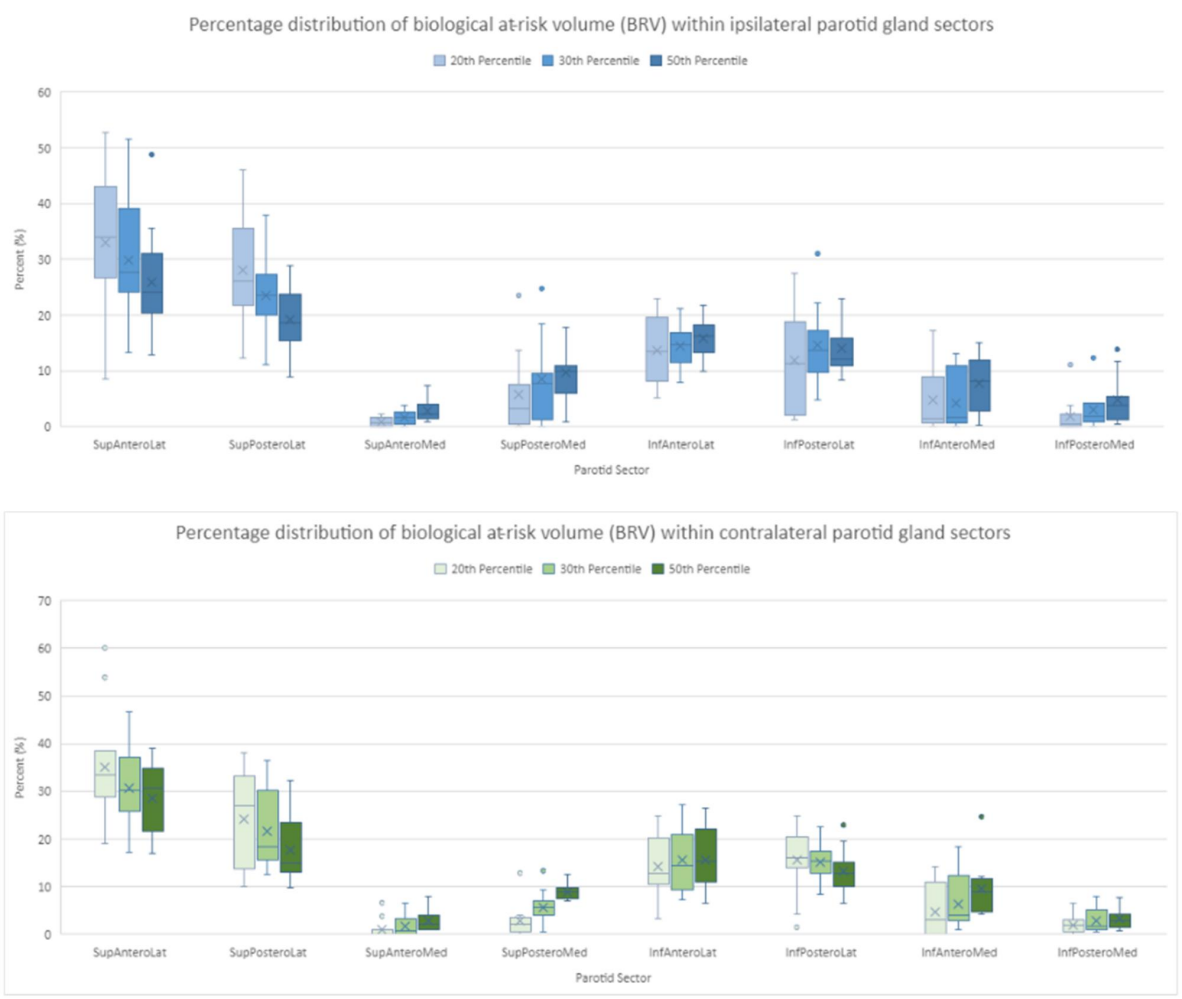
Box and whisker plots of the percentage distribution of biological at-risk volume across all anatomical sectors for ipsilateral and contralateral parotid glands.

**Figure 4.**
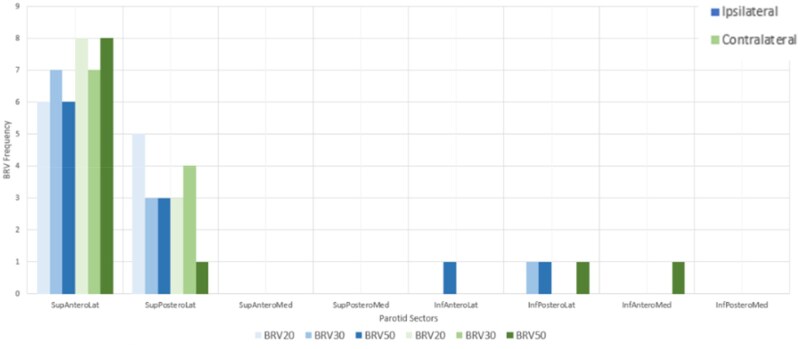
Frequency of the highest volume of biological at-risk volume within the parotid gland.

BRV20, BRV30, and BRV50 were analyzed across each anatomical sector to determine where the largest volume was located within the parotid gland. It was evident that the percentage distribution of BRV was mostly found in the SupAnteroLat and SupPosteroLat sectors across all percentiles in the ipsilateral and contralateral parotid glands (Figure 3). On average, the largest BRV20 volume occupied 36.2% of the ipsilateral BRV and 38.4% of the contralateral BRV, regardless of the predominant anatomical sector. BRV30 occupied 32% and 33.1% and the BRV50 occupied 27% and 30.6% of the ipsilateral and contralateral BRV’s respectively. The graph in Figure 4 displays the frequency of the largest distribution of BRV across all sectors, the majority is only seen in the medial aspect once.

### Statistical analysis

Pearson correlation tests comparing the volume of ADC percentile threshold contours within anatomical sectors of the parotids and their volumes was significant for the SupPosteroLat at the .01 level (2-tailed) across all BRV percentile thresholds in the ipsilateral parotid gland. This differs for the contralateral parotid, where the InfPosteroLat sector demonstrates the highest significant correlation. Pearson correlation values are detailed in Table 4.

**Table 4.** Pearson correlation values for whole biological at-risk volume (cc) and biological at-risk volume within each anatomical sector of parotid gland.

	Ipsilateral parotid gland			Contralateral parotid gland		
Anatomical sector	BRV20	BRV30	BRV50	BRV20	BRV30	BRV50
SupAnteroLat	0.762^a^	0.73^a^	0.760^a^	0.807^a^	0.796^a^	0.809^a^
SupPosteroLat	0.802^a^	0.844^a^	0.830^a^	0.766^a^	0.786^a^	0.740^a^
SupAnteroMed	−0.149	0.059	0.096	−0.72	−0.38	0.179
SupPosteroMed	0.073	0.201	0.311	0.038	0.775^a^	0.897^a^
InfAnteroLat	0.360	0.638	0.792^a^	0.656	0.646	0.670
InfPosteroLat	0.472	0.581	0.773^a^	0.947^a^	0.955^a^	0.916^a^
InfAnteroMed	−0.088	0.033	−0.41	0.009	0.087	0.285
InfPosteroMed	−0.111	0.106	−0.256	0.601	0.525	0.636

a Correlation is significant at .01 level (2-tailed).

### Qualitative observations

The BRV50 contour occupied approximately three quarters of the whole parotid gland volume. As such, this percentile threshold will be excluded in any future testing for dose optimization studies due to the comparable method in sparing the whole parotid gland.

It is evident that a consistent pattern emerged in the spatial distribution of the BRV distribution, with the majority located in the Superolateral regions, specifically the SupAnteroLat and SupPosteroLat sectors of both the ipsilateral and contralateral parotid glands. This clustering suggests the presence of a potentially hypercellular region within the parotid gland. Given the recurrent anatomical location of the BRV20 and BRV30 contours, this subvolume may correspond to a functionally critical region of the parotid.

## Discussion

This study investigated the use of ADC histogram analysis to generate a BRV of the parotid gland, providing insight into the hypercellular region of this major salivary gland. To our knowledge, it is the first to introduce the concept of a BRV using ADC percentile thresholds acquired on MRI-Sim to spatially map potentially functional subvolumes within the parotid gland. The goal being to generate an avoidance region that could be applied during radiotherapy optimization in HNC patients.

The results have demonstrated that the distribution of BRV20 and BRV30 contours is mostly situated in the superolateral aspect of the parotid gland. This is a key finding that shows a potential relationship between the 20th and 30th percentile ADC values and the BRV location within the parotid gland. It is indicative that this region could identify the radiosensitive area pertinent to parotid gland recovery.

Our findings are in accordance with previous research where Van Luijk 2009 discuss the irradiation of the caudal and cranial parts of the rat parotid gland. Their group have shown that when the cranial region was irradiated that the entire gland displayed effects of degeneration. In contrast, when the caudal region was irradiated, this degeneration was restricted to the exposed tissue, with signs of regeneration outside of the irradiated area. The superolateral location of the BRV in this study could be correlated with the cranial region as discussed in the rat models. It is indicated that the cranial or superior region of the parotid gland is linked to the stem cell location. In a later study, Van Luijk 2016 et al describe a sensitive subregion in the human parotid gland. The dose received to a region near the dorsal edge of the mandible where the first branching of Stensen’s duct occurs has been shown to predict post-treatment function more accurately than the mean dose to the whole gland. The evidence suggests a region dependent volume effect of the parotid gland where varying regions of sensitivity are prevalent, highlighting reduced confidence in the predictive power of an overall mean dose. Our present research is a continuation of this knowledge, whereby it has been shown that DW-MRI and ADC sequences can automatically identify a location within the parotid gland that is similar to what has been previously reported.

The largest distribution of BRV across all percentile thresholds is found in the most desirable area for use in dose optimization. The superolateral sectors of the parotid gland are usually superior and lateral to the primary tumour, providing better opportunities to reduce dose to the BRV, without compromising dose to target coverage. This aligns with split organ and superficial lobe sparing techniques that have been tested where anatomical landmarks have been used to create regions within the parotid gland.^14^^,^^17^ This approach has shown reductions in high grade xerostomia to 23%, 12 months post-radiation treatment^27^ as opposed to 38% in the well-known PARSPORT trial.^2^ It was also shown to be feasible to reduce the mean dose to the whole parotid gland, as well as to the newly defined segments within the gland. The BRV contours, as defined by ADC percentile thresholds, has successfully been used to identify the potential highly functional region within the parotid gland. The identified regions are predominantly found in the superolateral sectors which indicate that this method can be used to potentially individualize toxicity reduction in HNC patients. By reducing dose to the BRV there is potential to further improve QoL and xerostomia rates in HNC patients if optimal target coverage can still be achieved. Retrospective planning studies are required to ensure dose reduction is possible without compromising target coverage. If achievable, this could not only offer improvements to patient outcomes but also allows for the introduction of personalized cancer care. This warrants further investigation and reporting of toxicity and adverse reactions throughout HNC treatment, to see if there is any true links with ADC, parotid stem cells and the regeneration of this salivary gland.

Mapping the BRV distribution across each anatomical sector has allowed for the functional information to be grouped anatomically, as well as determine which sector holds the largest volume of BRV. This anatomical information has allowed us to review the location of the BRV distribution, but it has also provided a means to apply this clinically in departments that may not have access to an MRI-Sim. Although the patient specific functional data will not be used, by sparing the anterior and posterior superolateral sectors of the parotid gland using this division method, there is no reason that all HNC cannot reap the benefits of this research if a link can be strongly reported. The intersection point used to define the sectors was selected based on easily identifiable anatomical landmarks and included a standard reference point for dividing the parotid into superficial and deep lobes.^13^^,^^27^ It is noted that patient position, individual anatomy, and tumour location could affect the size of these sectors. If deemed necessary, a revision of this intersection point will be considered in a future prospective pilot study after data from a larger cohort has been reviewed.

Statistical analysis using the Pearson correlation method reveals varying levels of significance between the ipsilateral and contralateral parotid glands. As shown in Table 4, significant correlations were observed in the SupAnteroLat and SupPosteroLat sectors for both parotid glands for BRV20, BRV30, and BRV50. This finding is noteworthy as it indicates a potential spatial correlation in the BRV distribution within the parotid gland. However, it is important to clarify that this correlation reflects a proportional relationship between the whole BRV and the BRV within each anatomical sector, as the volume of the whole BRV contour increases, so does the volume of the BRV within the specified anatomical sector, independent of the percentage distribution. Specifically, in the contralateral parotid gland, a significant correlation was observed in the InfPosteroLat sector for each BRV, suggesting a proportional increase between the whole BRV and the BRV within this sector. This result may reflect the preserved integrity of contralateral tissue microstructure and cellularity since it is further away from the primary tumour. Although the physical volume of the BRVs between the ipsilateral and contralateral parotids were similar, the ADC map suggests that the contralateral gland function may be more consistent across patients. The presence of a linear relationship across both parotid glands is evident, as the significance for the BRV50 indicates that the lateral sectors tend to scale as the volume of the BRV contour increases. The clinical relevance of these correlations remain uncertain and requires validation with a larger sample size to determine any statistical significance.

DW-MRI and the associated ADC map were selected as the functional imaging modality in this study. These sequences are routinely acquired for HNC patients in our departmental MRI-Sim imaging protocols.^22^ Contrast is not required to attain any signal and acquisition time is relatively fast when compared to other functional methods. The initial work towards setting up RT specific MRI-Sim imaging protocols has allowed for the confidence in the results presented in this study. The consistent use of parameters including standardized *b* values has allowed for the accurate scrutinization of ADC values across all image data sets.^20^ ADC percentile thresholding has provided an opportunity for regional classification of the BRV across the parotid gland.

The process of identifying and sparing smaller, functionally critical regions is an innovative approach that could result in reductions in xerostomia rates. If target coverage can be maintained, specific dose constraints may be introduced to further drive optimization of the BRV, taking parotid-sparing techniques to the next level. Any future prospective studies will need to account for the impact of potential external separation changes and anatomical parotid changes which are common in this cohort of patients. Previous studies have reported increases in parotid gland ADC values throughout the course of RT, indicating underlying functional changes.^28^^,^^29^ Consequently, this raises concerns about the feasibility of applying the same ADC percentile thresholds throughout treatment. The adaptive application of parotid ADC values represents a potential area of future investigation, however, it would require careful consideration due to the influence of cumulative radiation dose on ADC values during treatment. While the small sample size is acknowledged as an obvious limitation, the results are promising and support the need for future prospective investigations on a larger scale to confirm the reproducibility of the BRV location in a clinical setting. The next step will be to determine whether the BRV can be effectively spared without compromising target coverage. If achievable, sparing the BRV could serve as a valuable approach during dose optimization, paving the way for personalized parotid-sparing RT planning in HNC patients.

## Conclusion

This study demonstrates the feasibility of deriving a BRV within the parotid gland using ADC percentile thresholding. It was found that the largest BRV distribution falls in the superolateral sectors, indicating a potential relationship with the 20th and 30th percentile ADC thresholds and the functionally critical region of the parotid gland. The favourable location of the BRV20 and BRV30 will allow for further sparing of the parotid gland during dose optimization which could lead to reductions in xerostomia, improved long term QoL and the development of personalized parotid-sparing RT techniques. These findings provide a foundation for future translational steps of this research which could lead to a novel opportunity for a prospective functional guided RT study in a larger cohort of patients in the near future.
